# The role of sensitization in musculoskeletal shoulder pain

**DOI:** 10.1590/bjpt-rbf.2014.0100

**Published:** 2015-08-07

**Authors:** John Borstad, Christopher Woeste

**Affiliations:** 1 Physical Therapy Division, School of Health and Rehabilitation Sciences, The Ohio State University, Columbus, OH, United States

**Keywords:** shoulder, pain, sensitivity, thresholds

## Abstract

**Introduction:**

Peripheral and central sensitization are neurophysiological processes that can prolong painful conditions. Painful shoulder conditions are often persistent, perhaps due to the presence of sensitization.

**Method:**

This manuscript summarizes six studies that have evaluated those with musculoskeletal shoulder pain for the presence of sensitization.

**Results:**

All six manuscripts report evidence of peripheral sensitization, while central sensitization was described in five of the studies. The chronicity of symptoms in subjects who were included in the studies is probably influencing this finding. The primary somatosensory test used to assess sensitization in these studies was Pressure Pain Threshold, a test for lowered nociceptive thresholds.

**Discussion:**

It appears that peripheral sensitization manifests consistently in those with musculoskeletal shoulder pathology, probably due to the inflammatory processes related to tissue injury. Central sensitization, while not universally present, was reported in a majority of the manuscripts. Because central sensitization is thought to be a key step on the pathway to chronic pain, evidence for its presence in those with shoulder pain is significant. Clinicians should expect the presence of sensitization with shoulder pathology and make appropriate choices about interventions so as not to exacerbate pain.

## Introduction

Musculoskeletal shoulder pain is one of the most common problems for which individuals seek medical care. Incidence rates as high as 30% have been reported^1^ and costs for the first six months of management are estimated at between $970 and $2,700 per episode^2^^,^ 3. The overall direct costs associated with treatment for shoulder dysfunction in the United States were reported as $7 billion in 2000^4^. Common shoulder impairments include pain, difficulty raising the arm overhead, impaired work and leisure participation, and disturbed sleep. Reducing shoulder impairments and their associated costs through effective conservative treatment is possible and worth pursuing. Unfortunately, consistently effective interventions have not emerged from the vast number of options available to clinicians. There are several possible explanations that may account for poor or modest treatment outcomes, one of which is sensitization.

Sensitization is a nervous system phenomenon that can occur in conjunction with pain^5^. With sensitization, normally innocuous input is perceived as painful due to increases in nociception^6^^-^ 8. Nociception is the activation of sensory organs by means of various forms of energy - such as mechanical, chemical, or thermal - at a level that suggests a risk of tissue injury^9^. When sensitization is present, the energy level required to activate nociceptors is decreased leading to increased pain perception. In addition to lowered nociceptive thresholds, increased pain perception can also result from prolonged activation of receptors, and/or activation of polymodal receptors. This broadening of nociception results in pain perception during activities or movements that would not normally be painful, and is called peripheral sensitization^10^. In most cases this is a normal response to injury as a mechanism to protect the injured tissue from further damage. Peripheral sensitization indicates that the expansion of nociception occurs in tissues innervated by the peripheral nervous system. With prolonged peripheral sensitization, central nervous system changes can also occur and result in central sensitization^11^^,^ 12. Central sensitization refers to altered neural thresholds in the spinal cord and/or reduced cortical inhibition of pain. It is hypothesized that central sensitization is a likely mechanism for the development of chronic pain syndromes^5^^,^ 13. Sensitization has been reported in many musculoskeletal conditions, including lateral epicondylalgia, patellar tendinitis, fibromyalgia, low back pain, temporomandibular pain, and shoulder pain^6^^,^ 8.

**Figure 1.**
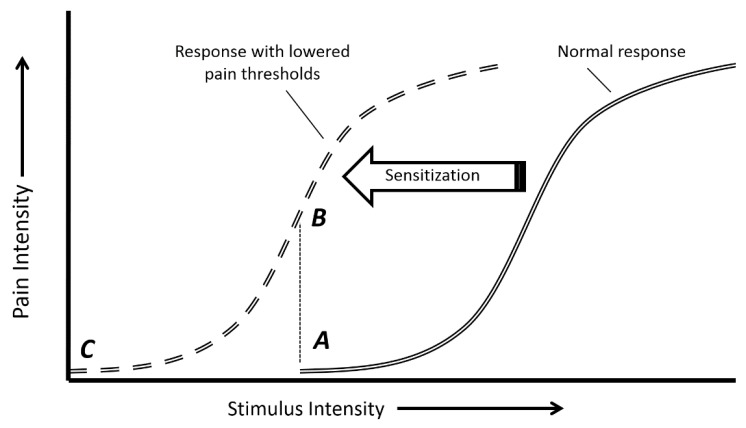
Hypothesized sensitization process. The normal response curve (double line) portrays the relationship between pain perception and stimulus intensity. In the presence of sensitization, this curve shifts to the left (double dashed line). (A) represents pain onset in the normal response condition; (B) represents hyperalgesia, in which a stimulus intensity that causes pain onset in the normal condition is perceived as more painful after sensitization; (C) represents allodynia, in which a stimulus intensity below that of normal onset is now perceived as painful.

Although the location of threshold changes differs between peripheral and central sensitization, the manifestation of both types of sensitization is a change in pain perception and/or intensity. Pain perception is the point at which a stimulus, such as mechanical pressure, becomes painful. When sensitization is present, this stimulus threshold is lowered such that a lower intensity stimulus is painful. This phenomenon is known as allodynia, or pain in response to a previously non-painful stimulus. Pain intensity, on the other hand, refers to the perceived magnitude of pain in response to the stimulus. In this case, when sensitization is present the same stimulus intensity results in greater levels of pain. This phenomenon is called hyperalgesia, or pain that is disproportionately high compared to the level of stimulus^9^. Allodynia and hyperalgesia are represented in Figure 1.

Determining the presence and extent of sensitization is accomplished through somatosensory testing^7^^,^ 14 ^-^ 17. Because pain is multidimensional and sensitization may manifest differently depending on the factors contributing to pain, a variety of somatosensory test outputs are possible. Rolke et al.^16^ describe a standardized quantitative sensory testing protocol involving seven tests of 13 sensory parameters, including tests for thermal detection, thermal pain thresholds, mechanical detection, and mechanical pain thresholds. One of the key goals of testing is to determine if sensitization is peripheral, central, or a combination of both. To accomplish this, testing is done both at the painful site and at non-painful sites. Changes in thresholds or sensitivity detected at non-painful sites indicate central sensitization. For example, in the patient with shoulder pain, increased sensitivity to thermal or mechanical input in the contralateral shoulder and/or leg would be interpreted as central sensitization.

For many clinicians, patients with subacromial impingement syndrome (SIS) are challenging to treat. SIS is challenging because the multi-factorial nature of its pathoetiology makes it difficult to determine the primary mechanistic factor(s)^18^^-^ 21. Because of these challenges, impairments may persist and allow sensitization to persist. Prolonged sensitization may further decrease the ability for interventions to be effective. In addition to unsuccessful or delayed recovery, the risk of progressing to chronic pain syndromes becomes greater the longer shoulder pain persists^7^^,^ 22. Several investigators have recognized this problem and have evaluated the sensitization phenomenon in groups of individuals with painful shoulders.

The purpose of this review was to summarize and to evaluate the outcomes of quantitative sensory testing on individuals with unilateral shoulder pain and to summarize what is known about the relationship between shoulder pain and sensitization. Table 1 notes the characteristics of those studies that evaluated the effect of shoulder pain on sensitization. It is clear from the relatively few studies that have quantified somatosensory thresholds in those with shoulder pain that sensitization was present. All studies reported the presence of peripheral sensitization, indicating that local nociceptive thresholds were consistently lowered with unilateral shoulder pain, while a majority of studies reported evidence for central sensitization^23^^-^ 28.

**Table 1.** Manuscript characteristics.

First Author	Type of Shoulder Pain	Number of Subjects	Controls (n)
Alburquerque-Sendin (2013)^23^	SIS	27	Asymptomatic age-, weight- and height-matched (20)
Paul (2012)^24^	SIS	31	Asymptomatic (31)
Gwilym (2011)^25^	SIS	17	Asymptomatic age- and sex- matched (17)
Hidalgo-Lozano (2010)^26^	SIS	12	Asymptomatic age-matched (10)
Hidalgo-Lozano (2013)^27^	USP	17	Asymptomatic Swimmers (18) Asymptomatic Athletes (15)
Coronado (2014)^28^	USP	58	Asymptomatic age- and sex-matched (56)

SIS: Subacromial Impingement Syndrome; USP: Unilateral Shoulder Pain.

### Quantitative sensory testing in individuals with unilateral shoulder pain

Pressure Pain Threshold (PPT) is an estimate of mechanical pain sensitivity. The PPT is the point at which individuals report that a gradually increasing force into soft tissue becomes painful. A lower PPT is indicative of decreased nociceptive thresholds to pain and signifies the presence of sensitization. PPT was quantified in five of the six studies that analyzed individuals with subacromial impingement syndrome or unilateral shoulder pain, and sensitization estimated by comparison to the PPT of control subjects. All five studies reported lower thresholds on the affected shoulder of SIS subjects when compared to thresholds for control subject shoulders^23^^,^ 24 ^,^ 26 ^-^ 28. Table 2 includes the evidence for sensitization in subjects with shoulder pain.

**Table 2.** Summary of evidence for sensitization: Evidence column describes how subjects with pain responded to somatosensory testing.

First Author	Peripheral Sensitization		Central Sensitization	
	Evidence	Comparison	Evidence	Comparison
Alburquerque-Sendin (2013)^23^	• Lower PPT in affected supraspinatus	• Control Group	• Lower PPT in unaffected supraspinatus	• Control Group
Paul (2012)^24^	• Lower PPT in affected shoulder	• Control Group	• Lower PPT in unaffected shoulder • Lower PPT in contralateral tibialis anterior	• Control Group • Control Group
Gwilym (2011)^25^	• Lower pain detection threshold affected side • Lower pain detection threshold affected side • Higher pain rating to sharpness affected side	• Unaffected side • Control Group • Unaffected side	• Unaffected shoulder not different	• Control Group
Hidalgo-Lozano (2010)^26^	• Lower PPT in affected shoulder muscles	• Control Group	• Lower PPT in ipsilateral tibialis anterior	• Control Group
Hidalgo-Lozano (2013)^27^	• Lower PPT in affected shoulder muscles	• Control Group	• Lower PPT in ipsilateral tibialis anterior	• Control Group
Coronado (2014)^28^	• Lower PPT in affected acromion • Lower PPT in affected shoulder • Lower PPT in affected side masseter	• Unaffected side • Control Group • Control Group	• Higher pain ratings to suprathreshold heat both sides • Lower PPT in unaffected shoulder	• Control Group • Control Group

PPT: Pressure Pain Threshold.

Gwilym et al.^25^ also assessed subjects with SIS for peripheral sensitization, but evaluated mechanical pain with sharpness detection rather than PPT. Subjects in this study reported when a sharp stimulus over the deltoid was perceived as sharp/painful, and by rating the level of pain experienced when a 256 mN probe was applied to the shoulder. Detection thresholds were significantly lower for the affected shoulder compared to the contralateral shoulder, indicating the presence of peripheral sensitization. The authors also reported that this presence of hypersensitivity to sharp stimuli led to poorer outcomes on the Oxford Shoulder Score three months after subacromial decompression surgery. Together with the PPT results, these studies confirm the presence of peripheral sensitization in those with SIS or unilateral shoulder pain (Table 2).

Several of these same studies evaluated PPT at sites remote to the affected shoulder such as the contralateral shoulder or tibialis anterior^23^^,^ 24 ^,^ 26 ^-^ 28. PPT's at remote sites were then compared to PPT's at the same anatomical site in control subjects. Lower PPTs were reported in the contralateral (unaffected) shoulder^23^^,^ 24 ^,^ 28, ipsilateral tibialis anterior^26^^,^ 27, and contralateral tibialis anterior^24^. Although not directly reported, it appears that mechanical pain thresholds of the contralateral (unaffected) shoulder in SIS subjects were not different than control subject thresholds in the Gwilym et al.^25^ study. The identification of lower PPT at tissues remote to the affected shoulder supports the presence of central sensitization in those with subacromial impingement syndrome and unilateral shoulder pain (Table 2).

### Relationship between shoulder pain and sensitization

From these six published analyses of somatosensory function, sensitization appears to be a regularly occurring phenomenon in individuals with unilateral shoulder pain. As noted earlier, peripheral sensitization represents a normal protective response to injury. As such, the presence of peripheral sensitization reported in these studies is not surprising. However, five of the six studies also reported indications of central sensitization in those with unilateral shoulder pain. This suggests that prolonged nociception from involved shoulder tissues could alter receptive fields in the spinal cord dorsal horn and/or the balance of descending pain inhibition and facilitation in those with SIS^6^. The rotator cuff muscles and tendons, particularly supraspinatus, are densely populated with nociceptors that likely contribute to central sensitization at the shoulder^29^.

Pain duration clearly impacts the transition from peripheral sensitization to central sensitization. Although the precise timing of this transition is unknown in humans, it is apparent that sensory signaling in the CNS becomes amplified by repeated peripheral input from injured or inflamed peripheral tissue over time^12^. The duration of pain reported in the studies reviewed was not consistently reported, but the shortest mean duration of pain was 8.5 months while the longest mean duration was 44.3 months. This suggests that the majority of subjects in these studies could be considered to have chronic pain, usually defined as pain lasting longer than three to six months. This limits the ability to make generalizations about those with acute shoulder pain or to better understand how the duration of injury affects both peripheral and central sensitization.

The implication of these findings is that conservative interventions for those with chronic unilateral shoulder pain are less likely to be effective or will require more resources. The consistent presence of central sensitization suggests that spinal cord processes and/or descending pain inhibition have been altered such that peripheral input may be perceived as nociceptive. This may influence the ability to successfully apply interventions that impact joint tissues such as stretching or strengthening exercises. It also suggests that simple functional activities, such as reaching overhead or personal care may exacerbate symptoms and diminish rehabilitation progress.

Because of the strong relationship between central sensitization and the development of chronic pain, it is imperative that the mechanisms by which peripheral sensitization transitions to central sensitization are discovered^6^. Duration of pain is only one factor in a process that includes interactions among sensory input, spinal cord signal processing, descending inhibition and facilitation, and complex biochemistry at each stage^30^. Preventing or slowing the progression to central sensitization has the potential to be extremely valuable in decreasing the burdens and costs associated with chronic pain. Similarly, discovering low-cost conservative interventions that may slow or reverse the transition from peripheral to central sensitization will be extremely valuable. Exercise and manual techniques such as massage or mobilization, when applied at the appropriate time and with the right intensity, may be able to modify aberrant peripheral input and positively alter central processing^31^. Early intervention for acute shoulder pain should also be promoted to potentially prevent the transition to central sensitization. However, aggressive exercise in the early stages of pathology may be detrimental if excessive or forceful movements trigger sensitized peripheral nociceptors and cause increased or prolonged pain. Clinicians must also be skilled at discerning and interpreting patient pain. Distinguishing soreness or minimal pain after therapeutic exercise from lasting and increased pain that is out of proportion to the activity is critical so that treatment modifications can be made to avoid further sensitization. Clinicians must also educate patients on any necessary activity restrictions or modifications so as not to feed into sensitization processes during the patients' work or recreational activities. Finally, effective interventions specifically targeting sensitization are not known at this time so studies that evaluate treatments designed to alleviate sensitization are needed.

## Conclusion

In summary, these studies suggest that both peripheral and central sensitization may be present in subjects with shoulder pain of musculoskeletal origin. Of the six studies reviewed, five determined that central sensitization was present based on PPTs or other somatosensory testing. This interpretation was made when lower PPTs were observed at anatomical sites remote to the affected shoulder when compared to controls. Overall, this review supports the idea that subjects with musculoskeletal shoulder pain may develop both peripheral and central sensitization as part of their pathology. This finding emphasizes the need for timely and effective interventions that resolve symptoms before sensitization becomes firmly established and more difficult to treat.

## References

[1] Bruls VE, Bastiaenen CH, de Bie RA (2013). Non-traumatic arm, neck and shoulder complaints: prevalence, course
and prognosis in a Dutch university population. BMC Musculoskelet Disord.

[2] Kuijpers T, van Tulder MW, van der Heijden GJ, Bouter LM, van der Windt DA (2006). Costs of shoulder pain in primary care consulters: a prospective
cohort study in The Netherlands. BMC Musculoskelet Disord.

[3] Dorrestijn O, Greving K, van der Veen WJ, van der Meer K, Diercks RL, Winters JC (2011). Patients with shoulder complaints in general practice: consumption of
medical care. Rheumatology (Oxford).

[4] Meislin RJ, Sperling JW, Stitik TP (2005). Persistent shoulder pain: epidemiology, pathophysiology, and
diagnosis. Am J Orthop (Belle Mead NJ).

[5] Arendt-Nielsen L, Fernández-de-Las-Peñas C, Graven-Nielsen T (2011). Basic aspects of musculoskeletal pain: from acute to chronic
pain. J Manual Manip Ther.

[6] Arendt-Nielsen L, Graven-Nielsen T (2011). Translational musculoskeletal pain research. Best Pract Res Clin Rheumatol.

[7] Graven-Nielsen T, Arendt-Nielsen L (2010). Assessment of mechanisms in localized and widespread musculoskeletal
pain. Nat Rev Rheumatol.

[8] Staud R (2011). Evidence for shared pain mechanisms in osteoarthritis, low back pain,
and fibromyalgia. Curr Rheumatol Rep.

[9] Coutaux A, Adam F, Willer J-C, Le Bars D (2005). Hyperalgesia and allodynia: peripheral mechanisms. Joint Bone Spine.

[10] Staud R (2010). Is it all central sensitization? Role of peripheral tissue nociception
in chronic musculoskeletal pain. Curr Rheumatol Rep.

[11] Nijs J, Van Houdenhove B, Oostendorp RA (2010). Recognition of central sensitization in patients with musculoskeletal
pain: Application of pain neurophysiology in manual therapy
practice. Man Ther.

[12] Woolf CJ (2011). Central sensitization: implications for the diagnosis and treatment of
pain. Pain.

[13] DeSantana JM, Sluka KA (2008). Central mechanisms in the maintenance of chronic widespread
noninflammatory muscle pain. Curr Pain Headache Rep.

[14] Arendt-Nielsen L, Yarnitsky D (2009). Experimental and clinical applications of quantitative sensory testing
applied to skin, muscles and viscera. J Pain.

[15] Rolke R, Magerl W, Campbell KA, Schalber C, Caspari S, Birklein F (2006). Quantitative sensory testing: a comprehensive protocol for clinical
trials. Eur J Pain.

[16] Rolke R, Baron R, Maier C, Tölle TR, Treede RD, Beyer A (2006). Quantitative sensory testing in the German Research Network on
Neuropathic Pain (DFNS): standardized protocol and reference
values. Pain.

[17] Maier C, Baron R, Tölle TR, Binder A, Birbaumer N, Birklein F (2010). Quantitative sensory testing in the German Research Network on
Neuropathic Pain (DFNS): somatosensory abnormalities in 1236 patients with
different neuropathic pain syndromes. Pain.

[18] Braman JP, Zhao KD, Lawrence RL, Harrison AK, Ludewig PM (2014). Shoulder impingement revisited: evolution of diagnostic understanding
in orthopedic surgery and physical therapy. Med Biol Eng Comput.

[19] Goldberg SS, Bigliani LU (2006). Shoulder impingement revisited: advanced concepts of pathomechanics
and treatment. Instr Course Lect.

[20] Ludewig PM, Braman JP (2011). Shoulder impingement: biomechanical considerations in
rehabilitation. Man Ther.

[21] Seitz AL, McClure PW, Finucane S, Boardman ND 3rd, Michener LA (2011). Mechanisms of rotator cuff tendinopathy: intrinsic, extrinsic, or
both?. Clin Biomech (Bristol, Avon).

[22] Fornasari D (2012). Pain mechanisms in patients with chronic pain. Clin Drug Investig.

[23] Alburquerque-Sendín F, Camargo PR, Vieira A, Salvini TF (2013). Bilateral myofascial trigger points and pressure pain thresholds in
the shoulder muscles in patients with unilateral shoulder impingement syndrome: a
blinded, controlled study. Clin J Pain.

[24] Paul TM, Soo Hoo J, Chae J, Wilson RD (2012). Central hypersensitivity in patients with subacromial impingement
syndrome. Arch Phys Med Rehabil.

[25] Gwilym SE, Oag HC, Tracey I, Carr AJ (2011). Evidence that central sensitisation is present in patients with
shoulder impingement syndrome and influences the outcome after
surgery. J Bone Joint Surg Br.

[26] Hidalgo-Lozano A, Fernández-de-las-Peñas C, Alonso-Blanco C, Ge HY, Arendt-Nielsen L, Arroyo-Morales M (2010). Muscle trigger points and pressure pain hyperalgesia in the shoulder
muscles in patients with unilateral shoulder impingement: a blinded, controlled
study. Exp Brain Res.

[27] Hidalgo-Lozano A, Fernández-de-las-Peñas C, Calderón-Soto C, Domingo-Camara A, Madeleine P, Arroyo-Morales M (2013). Elite swimmers with and without unilateral shoulder pain: mechanical
hyperalgesia and active/latent muscle trigger points in neck-shoulder
muscles. Scand J Med Sci Sports.

[28] Coronado RA, Simon CB, Valencia C, George SZ (2014). Experimental pain responses support peripheral and central
sensitization in patients with unilateral shoulder pain. Clin J Pain.

[29] Dean BJF, Gwilym SE, Carr AJ (2013). Why does my shoulder hurt? A review of the neuroanatomical and
biochemical basis of shoulder pain. Br J Sports Med.

[30] Thomas Cheng H (2010). Spinal cord mechanisms of chronic pain and clinical
implications. Curr Pain Headache Rep.

[31] Bialosky JE, Bishop MD, Price DD, Robinson ME, George SZ (2009). The mechanisms of manual therapy in the treatment of musculoskeletal
pain: a comprehensive model. Man Ther.

